# Ethical considerations of the duty to care and physician safety in the COVID-19 pandemic

**DOI:** 10.1017/cem.2020.376

**Published:** 2020-04-24

**Authors:** Francis Bakewell, Merril A. Pauls, David Migneault

**Affiliations:** * Department of Emergency Medicine, The Ottawa Hospital, Ottawa, ON; † Medicine, Ethics, and Humanities Program, Department of Innovation in Medical Education, Faculty of Medicine, University of Ottawa, Ottawa, ON; ‡ CAEP Bioethics Committee; § Health Sciences Centre, Max Rady College of Medicine, Department of Emergency Medicine, University of Manitoba, Winnipeg, MB; ¶ Vancouver General Hospital, Vancouver, BC; # BC Children's Hospital, Vancouver, BC; ∥ UBC Urgent Care Centre, Vancouver, BC; ** Vancouver Coastal Health, Vancouver, BC; †† UBC Department of Emergency Medicine, University of British Columbia, Vancouver, BC

**Keywords:** Ethical obligations, COVID-19, pandemic, physician safety

## INTRODUCTION

Some of the most frightening stories that have emerged from the coronavirus disease 2019 (COVID-19) pandemic have been of overwhelmed physicians caring for patients despite a lack of adequate personal protective equipment (PPE). During a pandemic, questions naturally arise around the balance between a physician's duty to provide care to patients, their obligations to protect their family and loved ones, and their right to protect their own health.

Any decision-making frameworks we use in these exceptional times should consider the traditional principles of medical ethics, but also must reflect the core principles of public health ethics: respect, the harm principle, fairness, consistency, using the least coercive and restrictive means, working together, reciprocity, proportionality, flexibility, and procedural justice.

## COMPETING OBLIGATIONS

The World Health Organization outlines the obligations of health care providers in the context of a pandemic as falling under three main categories: moral, professional, and legal.^1^

## MORAL OBLIGATIONS

Several ethical principles and professional responsibilities establish the importance of providing care to our patients, even when it is inconvenient or there is risk. These include a fiduciary relationship with our patients, a duty to care, and the ethical principles of beneficence and nonmaleficence.

At the same time, physicians have a right to protect their own health and minimize personal risks. They have a right to protect their families and loved ones. Physicians also have a special obligation to preserve their ability to care for current and future patients.

Relevant public health ethics considerations include that physicians should be willing to take on additional risks if others in society are doing so, and they have specialized skills that are required at this time. This may be in tension with the principle of reciprocity (those who take additional risks or suffer harm should receive compensation or special consideration) and of using the least coercive or restrictive means.

Balancing these obligations and rights involves determining the relative importance of each and minimizing the restrictions that one imposes on another. A physician must justify any discharge of their ethical duty to care in relation to their participation in a specific patient care activity (or activities) that pose intolerable and unmitigable risk of certain and significant harm, and in relation to their own unique personal circumstances.^2^ It should also be noted that there are conceivable situations where the risk of harm is so great for all providers, that continuing to provide care transcends an obligation and becomes a moral ideal (i.e., praiseworthy, but not expected).

## PROFESSIONAL OBLIGATIONS

### Canadian Medical Association

The Canadian Medical Association (CMA) has a 2008 policy (*Caring in a Crisis: The Ethical Obligations of Physicians and Society during a Pandemic*) which recognizes that “physicians and other health care providers will be expected to put themselves in harm's way, and to bear a disproportionate burden of the personal hardships associated with a pandemic.”^3^ However, the policy also emphasizes the reciprocal obligations of society to ensure that the health and well-being of providers is maximized while they take on this additional risk. A recent CMA statement (“Framework for Ethical Decision Making During the Coronavirus Pandemic”) emphasizes the importance of preferentially providing resources and equipment to front-line providers to preserve their ability to care for patients, and to recognize the added risks they are assuming.^4^

### Medical regulatory authorities

Most regulatory authorities have recognized that physicians cannot and should not be made to work in conditions that expose them to significant risks. At the same time, they are asking physicians to keep working in ways that will ensure patients have access to needed care. A good example of this balance is found in the College of Physicians and Surgeons of Ontario (CPSO) policy on *Public Health Emergencies*, updated in 2018. They clearly state that physicians have an obligation to provide care in pandemics, but outline that physicians who are unable to provide direct care for health reasons “must engage in indirect activities that support the response effort during public health emergencies.”^5^

### Canadian Association of Emergency Physicians

The Canadian Association of Emergency Physicians (CAEP) has made the following statements regarding exceptions to the duty to care during the COVID-19 pandemic: “We believe that physicians over the age of 60, particularly those with medical co-morbidities, must carefully weigh the risks to their own health in working in the ED during the COVID pandemic,”^6^ and “depending on the local circumstances, the risk of these physicians (over the age of 60, with chronic medical conditions, or immunocompromise) should be considered in work assignments while balancing against the overall department status and risk.”^7^

## LEGAL OBLIGATIONS

While contractual obligations will vary from institution to institution, there are several noncontractual legal obligations regarding medical care in a pandemic, and to what extent physicians can refuse work they believe is unsafe.

The legal duty of physicians to care for patients is primarily a matter of case law and professional standards of care in Canada, rather than specific legislation.^8^ While the duty to care is well-established for pre-existing physician–patient relationships, whether or not physicians have a duty to care for people who are not yet their patients is less clear. Quebec is the only province with specific legislation that mandates all people have a duty to come to the aid of someone in peril. Case law also suggests there is a legal duty to care for patients you have not yet met if you are specifically designated to care for that group of people (e.g., an emergency department physician or a physician on call).^9^

Some emergency physicians are employees of a hospital rather than independent contractors, and each province will have legislation that outlines the circumstance under which they may be legally allowed to refuse what they believe is unsafe work. As well, many provinces have emergency legislation that provides the authority to designate health care providers as “essential services,” thereby legally compelling their time and work, although this would likely be a last resort if faced with a shortage of available providers.^10^

The Canadian Medical Protective Association (CMPA) has said that the professional obligations and legal principles that usually apply to all physicians continue in the context of COVID-19, that physicians have a legal duty to ensure everything they do for their patients meets the standard of care of a reasonably competent physician in similar pandemic circumstances, and that physicians still must meet the obligations set out in policies by their respective colleges.^11^

## RECIPROCAL RESPONSIBILITIES

Along with the professional moral obligations of providers, there are reciprocal obligations for governments and institutions to minimize risks to providers to the greatest extent possible, for example, by ensuring adequate infection control measures, preventative measures (e.g., PPE), and prioritizing access to care should providers become sick.

**Recommendations:**

1) The COVID-19 pandemic has increased potential risks for emergency physicians, especially given the undifferentiated nature of our patient population; however, in a fully staffed department, with appropriate equipment and training, the risks remain relatively small and otherwise healthy emergency physicians (under a certain age) should be expected to fully participate in the patient care activities of their emergency department.
2) A physician must justify any discharge of their ethical duty to care in relation to their participation in a specific patient care activity (or activities) that pose intolerable and unmitigable risk of certain and significant harm, and in relation to their own unique personal circumstances. They should still be expected to contribute in other nonclinical ways.
  a. Doctors with any well-recognized increased risk of harm (e.g., advanced age, immunocompromise) should be allowed to opt out of direct patient care due to their higher risk for a bad outcome if they contract COVID-19, but should be permitted to continue providing care if they choose.
  b. Other requests to opt out of direct patient care should be assessed on an individual basis. Such an assessment should include the merits of the request, the availability of physician resources, generalizability (i.e., could all other similar requests be accommodated), and to what degree this accommodation would increase the risk of colleagues who continue to provide direct care. Some arguments for special consideration may include compelling personal circumstance (e.g., single parent, sole care-giver) or unique professional skills/abilities (e.g., department head, director of emergency medical service [EMS]).
3) In situations where PPE is in short-supply or unavailable, the relative strength of the duty to care may be significantly weakened. However, there are other ethical and professional obligations to balance, including our fiduciary relationship with our patients, the need for our specialized skills, and maintaining solidarity with our colleagues and other health care providers. Appropriate responses to a shortage of PPE should focus on institutional and government responsibilities, rather than the withdrawal of care to patients.
4) The reciprocal responsibilities that institutions and governments have to emergency physicians working in a pandemic need to be clearly articulated and a firm commitment made to fulfilling them. These include:
  a. Ensuring adequate PPE.
  b. Instituting clear policies and procedures in the event that PPE shortages occur (this should include prioritizing resources for high-risk areas and outlining acceptable alternatives to ideal PPE).
  c. Ensuring appropriate processes to prevent transmission of COVID-19 and provide care to front-line workers who need it.
